# Early Memories of Individuals on the Autism Spectrum Assessed Using Online Self-Reports

**DOI:** 10.3389/fpsyt.2016.00079

**Published:** 2016-05-02

**Authors:** Vera Zamoscik, Daniela Mier, Stephanie N. L. Schmidt, Peter Kirsch

**Affiliations:** ^1^Department of Clinical Psychology, Central Institute of Mental Health, Mannheim and Medical Faculty Mannheim, Heidelberg University, Mannheim, Germany

**Keywords:** autism, HFA, autobiographical memory, sensory system, language development

## Abstract

“When I was one and a half years old, I was on a ferry lying on red seats” – while several autobiographical accounts by people with autism reveal vivid memories of early childhood, the vast amount of experimental investigations found deficits in personal autobiographic memory in autism. To assess this contradiction empirically, we implemented an online questionnaire on early childhood events to compare people on the autism spectrum (AS) and non-autistic people with respect to their earliest autobiographical episodic memories and the earliest semantic *know event* as told by another person. Results indicate that people on the AS do not differ from non-autistic people in the age of their earliest *know events* but remember events from an earlier age in childhood and with more sensory details, contradicting the assumption of an overall deficit in personal episodic memory in autism. Furthermore, our results emphasize the supporting influence of language for memory formation and give evidence for an important role of sensory features in memories of people on the AS.

## Introduction

Autism is a pervasive developmental disorder in which some of the important core processes required for memory formation are impaired. Specifically, memory formation is influenced by difficulties in social interaction and communication, problems in the formation of new scripts, a tendency to display repetitive behaviors, and often narrow interests, which are typical characteristics in autism.

A number of studies on episodic memory report deficits in people with autism [e.g., Ref. (1–7)], which seem to be augmented in males in comparison to females, possibly due to differences in verbal fluency (8). Mostly, these studies included direct social interaction, referred to predefined events or contexts, or asked for autobiographical memories formed later in life than in early childhood. Interestingly, studies differentiating between semantic *know* and episodic *remember events* have shown that only the episodic but not the semantic autobiographical memory is impaired in autism (4, 9). For example, Tanweer and colleagues reported that not the entire autobiographical memory is affected in autism (*know events* are preserved), but only those aspects that can be related to the ability to relive one’s past, known as autonoetic awareness (*remember*) (9). The authors concluded that a deficit in autonoetic awareness, as well as a broad lack of specificity (which is a lack of specific information on time and place), causes autobiographical deficits in autism. Other authors attributed deficits in autobiographical memory in autism to a failure in the development of self-identity (4) or to impairments in Theory of Mind and working memory (5). Also, memories of people with autism were shown to include fewer social and emotional details (10).

Interestingly and contrariwise to the mentioned experimental studies, some individuals with high-functioning autism seem to be able to recall personal events from a very young age [e.g., Ref. (11–13)]; and moreover, these memories are rich in sensory detail. Not only are sensory features included in the new diagnostic criteria of the autism spectrum disorder [ASD; (14)] but also has sensory perception been reported to be atypical in 69–100% of individuals with autism [e.g., Ref. (15–19)]. Since the probability of encoding increases with a stronger involvement in a particular situation (20), one could assume that individuals with autism are better in memorizing sensory details than non-autistic individuals. Concordantly, according to the intense world theory by Markram and Markram (21), individuals with autism perceive the world more intensely than non-autistic individuals, due to overactive brain circuitry. The authors propose that a hyperactivation in these brain circuitries could account for hyper-perception, hyper-attention, hyper-emotionality, and even hyper-memory in autism. Hence, there seems to be a contradiction between the findings of experimental studies asking mainly for specific memories and free reports of autobiographical memories in autism. From this perspective, it seems possible that people with autism even have improved personal autobiographical memories in free recall or with regard to (sensory) details.

One feature in autism that could be related to memory formation is altered language acquisition. The development of language and narrative structures enables children to encode memories linguistically, which in turn improves retrieval of autobiographical memories in adulthood using similar pathways (22), while sensory memories appear to become less important over a person’s lifetime. Studies found linguistic differences in autism with regard to several features. In a study with different narrative tasks, teenage children with ASD used language that is less descriptive and less grammatically complex (23). In another study, the narratives of adults with high-functioning autism or Asperger syndrome were less well organized and less cohesive compared to the control group (CG), even though the plot of the story was equally well comprehended (24). Losh and Gordon (25) reported differences in narrative ability in individuals with high-functioning ASD only during semi-structured conversation, which included narrative recall, but not when narrating the story from a picture book. The authors suggest that the picture book might be helpful in engaging the strong visual spatial skills of people with autism, thereby providing coherence. The conversation with the experimenter during the narrative recall, on the other hand, may be more difficult due to the social interaction.

Language and narrative skills are also important for the formation of a self-concept as they facilitate abstraction and reflection. During the process of language acquisition, children begin to form a remembered and cognitive self [e.g., Ref. (26)]. On the basis of this cognitive self, the ability arises to relate current and remembered events to the actual self and to attach importance to events, leading to stronger encoding and integration of memories in an associative network (26). Conway pointed out that memory and the self are interconnected, in that autobiographical memories shape the self, and the self-concept together with associated personal goals shapes the types of memories likely to be recalled (27). In autism, the self-concept seems to be atypical, considering the reduced awareness of own emotions or mental states (28). Supporting the assumption of a diminished self-concept, individuals with ASD show less self-referential processing than control subjects in experimental settings (29, 30). Self-referential thoughts are closely linked to activity in the medial prefrontal cortex [mPFC; (31)]. The mPFC also plays a central role in the unifying theory by Shalom (32), who highlights the importance of the mPFC and its role at the integrative level of different processing levels, suggesting its responsibility for the atypical characteristics in autism. Besides facilitating long-term storage of memories, the mPFC may help to integrate different aspects of an experience by strengthening synapses between relevant neurons (33). Difficulties of individuals with ASD in integrating different aspects into a coherent composition were also shown in studies on language and narrative skills. Lind and colleagues (34) proposed that impaired episodic memory function may be due to reduced scene construction ability, which is independent of general narrative skills.

We used a well-established questionnaire to assess early *know* and *remember events* in free recall (35, 36) to investigate effects of both event types and also possible interaction effects of group and event. We applied this questionnaire for the first time to people on the autism spectrum (AS). Additionally, we measured autistic traits by means of the autism spectrum quotient [AQ; (37)]. In order to better meet the special needs of this group, we circumvented any social interaction and instead set up an online study. Based on autobiographical accounts of people with autism and the assumptions made in the intense world theory (21), but in contrast to many experimental investigations of personal autobiographical memory in autism, we hypothesized that people on the AS remember earlier childhood events when having free choice which memory to recall and no cues are given. In addition, we examined the influence of two specific factors that might contribute to early memory formation: sensory processing and language acquisition.

## Materials and Methods

This study was performed in line with the Declaration of Helsinki, and the experimental protocols were carried out in accordance with the recommendations of the University of Mannheims’ Ethics Committee with written informed consent from all subjects.

The study was conducted *via* an online survey programed with the software testMaker (38) hosted by the Department Psychology III at the University of Mannheim, Germany. To reach people with autism, the link to the study was posted in four Internet forums dealing with autism and, to reach controls, the link was distributed *via* other forums and online platforms. As an incentive to participate in the study, a lottery for four 10-Euro book vouchers was offered.

First, participants answered items consisting of demographic questions about age, sex, German language skills, and education (inclusion criteria: legal age, a high school degree, and good German language skills). To investigate the relation between earliest memory and language, participants were asked for their age of language acquisition. Due to the online nature of the study, no diagnoses could be given or confirmed. However, in an attempt to learn about the history of autism diagnosis, participants were asked to report professional autism diagnosis and further neurological or psychiatric conditions. After assessing this information, the AQ and the questionnaire on early childhood events were followed.

Participation in this study required the ability to read and to write. Furthermore, all participants reported having at least a high school degree and word count of reported *remember events* did not differ between groups (see below). Hence, we consider all people able to complete the study.

### Adult Autism Spectrum Quotient

To estimate the number of people with high autism traits in the sample and to verify self-reported diagnoses all participants completed the Adult AQ by Baron-Cohen and colleagues (37). This questionnaire is valid for ages 16 years and above. The German translation by Freitag^1^ was used with item 17 and 27 in the translation of Dammann^1^. Validity and test–retest reliability of the AQ are high (37). Thus, the AQ is considered a reliable self-assessment screening instrument for autistic traits (37, 39).

### Participants

The “autism spectrum” (AS) group included people with high autism traits who scored 26 or higher [as suggested for clinical samples by Woodbury-Smith et al. (39)] in the Adult AQ and had a self-reported professional autism diagnosis. The CG consists of those participants without a self-reported autism diagnosis and an AQ score below 26. In the total sample of 317 people, AS participants were significantly older than control participants, so we decided on not including the whole CG but instead matched groups by age, sex, and education. Final sample size was *N* = 166. The AQ score differed significantly between groups [*F*(1,165) = 1,248.10, *p* < 0.001, *f* = 2.76]. For a summary of sex, age, education, AQ score, and self-reported neurological and psychiatric diagnoses beyond autism, see Table 1.

**Table 1:** **Demographic variables (sex, age, and education), AQ score, and occurrence of self-reported psychiatric and neurological diagnoses beyond autism**.

	AS group (*n* = 83)	Control group (*n* = 83)
Sex: male/intersex/female	31/1/51	32/0/51
Mean age in years (SD)	36 (10)	36 (12)
Mean education in years (SD)	13 (3)	13 (3)
Mean AQ score (SD)*	42 (5)	15 (5)
Depression*	16	5
AD(H)D*	11	0
Social anxiety	2	0
Personality disorder	1 schizoid	1 borderline
Tourette syndrome	1	0
Epilepsy	4	1

*AS, autism spectrum*.

*More than one diagnosis mentioned by one individual is possible*.

*indicates significant differences between groups (*p* < 0.05).

### Questionnaire on Early Childhood Events

Early childhood memories and sensory characteristics were assessed with the questionnaire developed by Bruce and colleagues (35, 36). The questions about *know* and *remember events* with examples, the estimations of ages, the certainty of age estimation (35), and the rating scales of characteristics of the *remember events* (36, 40) from the original English questionnaire were translated into German by the authors (translation provided upon request). In this questionnaire, people are asked to report a total of two autobiographical events from childhood: their earliest *know* and earliest *remember event* (including remembered fragment memories). Additionally, participants indicated the estimated ages when the reported events had happened and a confidence judgment regarding these age estimations. A description of the two kinds of events and short examples were provided to the participants. The age of events was assessed *via* the item “Please specify your age at event occurrence as accurately as possible [in years and months]:” including a box in which participants could freely enter the specific age. When participants knew that an event occurred, but could not relive any details relating to it, these events are referred to as *know events*. These events are based on external sources such as photographs or stories told by friends and family. In their description of the *know events*, participants had to indicate the source of their memory. *Remember events* are memories that are pure personal recollections specific to time and place; these events could be relived by the participant and relied on no other sources. In addition to freely describing each event in a short paragraph, participants rated the amount of several details of the *remember events* with 20 items (35, 36). We focused our analyses on eight of these items for the investigation of the influence of the sensory system and language on the *remember events*. These items comprised questions about the amount of visual, acoustic, olfactory, tactile, and taste components, richness of details of the event, and how often participants had talked or thought about the event afterward (e.g., “My recollection of the personal event I just described includes visual details:”) on a scale from one (*none/never*) to seven (*very much*). Report order of the two event types was counterbalanced across participants: half of the sample first recounted a *remember event* and corresponding questions and the other half a *know event*.

Prior to the rating of the events, data from individuals not meeting the inclusion criteria were removed (e.g., participants without high school degree). The reports were randomly ordered, and their identity (*remember* or *know event*, described by an AS individual or not) was concealed. Each event was independently evaluated by two of the authors as to whether it was a *remember event*, a *know event*, or neither. Inter-rater reliability was good (Cronbach’s α = 0.89). Description of repeated events (like “I was told I was crying a lot when I was a baby”), the autobiographical fact “I was born,” prenatal memories, and not clearly relatable events (e.g., no source was mentioned in *know events*) were excluded from data. Furthermore, two authors blindly counted and rated several details in the description of the *remember events*. The ratings were partly adapted from Levine and colleagues (41). In the present study, we recorded the overall word count and the amount of words related to the self (I, mine, etc.), social in-group (we, our, etc.), other persons (sister, his, etc.), and things (teddy, carpet, etc.). We also rated the *remember event* descriptions with regard to the amount of social and sensory details and number of different senses involved. In addition, the number of references to time (e.g., in the morning, fourth birthday), place (e.g., house of grandparents, France), and thoughts and emotions (such as proud or sad) as well as whether the memory was a fragment memory or not was rated. Inter-rater reliability was very good (Cronbach’s α = 0.99). Word counts were standardized to 40 words to adjust for slightly different but not significant word counts between groups (please see “External Rating of the *Remember Events*” for total word count numbers). Please see the Appendix for a selection of *remember events* from both groups.

## Results

*Know events* of 53 participants (24 CG, 29 AS) out of the 166 were not used for analyses, because no source was mentioned in the description. In some cases [not significantly different between groups, *F*(1,165) = 0.10, *p* = 0.754, *f* = 0.03], there was no full stop at the end of the description of the *remember event*, and we assume that occasionally few words were lost in the export procedure. This did not affect any results.

The order of the description of *remember* and *know events* did not affect results of estimation of the age at the time of the event [*remember*: *F*(1,165) = 0.05, *p* = 0.816, *f* = 0.00; *know*: *F*(1,165) = 0.47, *p* = 0.493, *f* = 0.06]. In addition, confidence judgments of the event ages did not differ between report orders [*remember*: *F*(1,165) = 0.00, *p* = 0.993, *f* = 0.00; *know*: *F*(1,165) = 0.13, *p* = 0.717, *f* = 0.03].

Confidence judgments did not differ between groups for *remember events* [*F*(1,165) = 0.14, *p* = 0.707, *f* = 0.03] but they did for *know events* in which the CG was less certain [*F*(1,165) = 7.76, *p* = 0.006, *f* = 0.26]. Self-reported age of language acquisition differed slightly but not significantly between groups [*F*(1,155) = 3.01, *p* = 0.085, *f* = 0.14]. Since the number of self-reported professional diagnoses of depression [*F*(1,165) = 6.79, *p* = 0.010, *f* = 0.20] and AD(H)D [*F*(1,165) = 12.53, *p* = 0.001, *f* = 0.28] differed significantly between groups (see also Table 1), these features were included as covariates in all following ANCOVAs to be able to detect and control for possible effects of those variables. In some cases, the covariates had a significant effect: in participants with depression, overall word count was greater, the number of thoughts, and different senses involved in *remember events* was higher [word count: *F*(1,165) = 5.40, *p* = 0.021, *f* = 0.18; thoughts: *F*(1,165) = 7.01, *p* = 0.009, *f* = 0.21; senses: *F*(1,165) = 5.80, *p* = 0.017, *f* = 0.19]. Furthermore, sensory details were reported less often in people with AD(H)D [*F*(1,165) = 5.88, *p* = 0.016, *f* = 0.19]. Thus, in the following, statistics for these details are additionally reported for the sample without those participants who reported depression and/or AD(H)D diagnosis. Except for the above mentioned small differences, excluding all participants with self-reported depression and/or AD(H)D diagnoses (new *n*: AS 58, CG 78, age, and sex did not differ significantly) did not change the pattern of results. Therefore, we decided to include all participants in the analyses to make results more representative.

### Ages of Described Events

**Figure 1:**
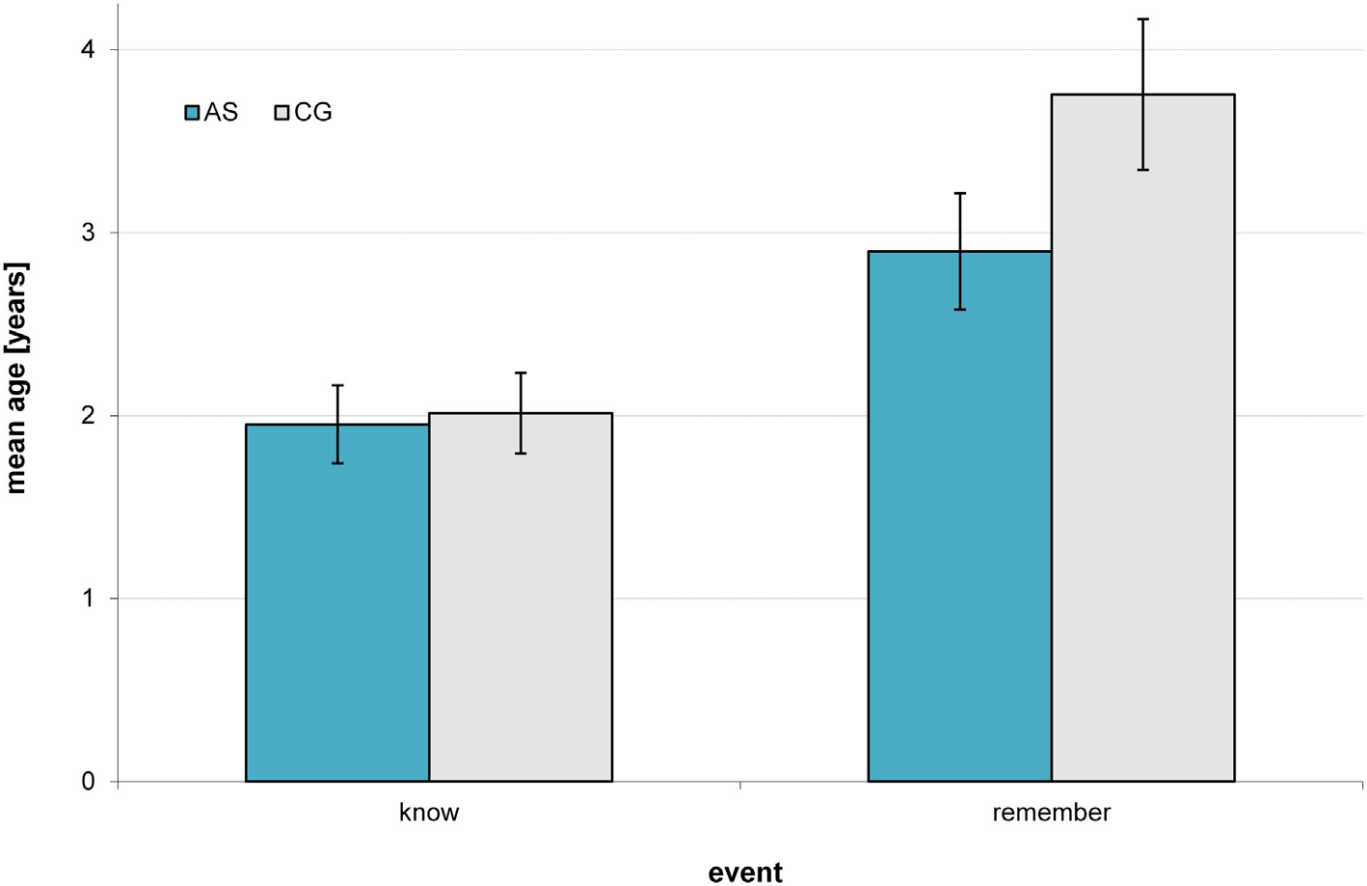
**Mean age of the earliest events in participants on the autism spectrum (AS) and matched controls (CG)**. Error bars indicate ± SE; mean age of *know events* did not differ between groups, mean age of *remember events* of the AS group (*M* = 2.90, SD = 1.56) differed significantly compared to the one of the CG (*M* = 3.76, SD = 1.41); for further details, see text.

In a mixed design ANCOVA with event (*remember/know*) and group (AS/CG) as factors and reported ages at time of the event as a dependent variable, we found no main effect of group [*F*(1,109) = 1.48, *p* = 0.226, *f* = 0.11; Levene’s test for homogeneity was not significant: *p*s > 0.300], but a significant main effect of event [*F*(1,109) = 45.93, *p* < 0.001, *f* = 0.65], and a significant interaction of group and event [*F*(1,109) = 4.17, *p* = 0.044, *f* = 0.20]. Participants on the AS reported significantly earlier ages for *remember events* than the control participants [*F*(3,165) = 9.34, *p* = 0.003, *f* = 0.24], while groups did not differ in the mean age of *know events* [*F*(3,112) = 0.02, *p* = 0.890, *f* = 0.00; Figure 1].

### Sensory Characteristics of the *Remember Events*

People on the AS remembered more sensory details from their earliest remembered childhood event. Over all senses including visual detail, smell, sound, touch, taste, and richness of details, this relationship was not significant [AS 22.33 ± 7.01, CG 20.00 ± 6.50, *F*(3,165) = 2.19, *p* = 0.141, *f* = 0.11]. However, the AS group reported significantly more visual detail, sound, and richness of details [*F*(3,165) = 10.14, *p* = 0.002, *f* = 0.25], whereas depressed participants reported to remember more details related to smell, touch, and taste [*F*(3,165) = 5.98, *p* = 0.016, *f* = 0.19].

In a correlation analysis, age of earliest *remember event* was related to the amount of reported visual detail in the AS group, but not in the CG [AS: *r*(83) = −0.31, *p* = 0.004; CG: *r*(83) = −0.17, *p* = 0.130; difference: *z* = 0.95, *p* = 0.343].

### Language Acquisition and Language Characteristics of the *Remember Events*

People on the AS reported talking less often about their *remember events* [*F*(3,164) = 13.25, *p* < 0.001, *f* = 0.29], while both groups thought equally often about them [*F*(3,164) = 1.03, *p* = 0.312, *f* = 0.08]. Interestingly, however, people reported earlier ages for *remember events* the more often they talked about them [AS: *r*(83) = −0.27, *p* = 0.015; CG: *r*(82) = −0.22, *p* = 0.046]. In addition, the age at which participants in the AS group reported first speaking showed a negative correlation with the amount of talking about their earliest *remember events* [*r*(78) = −0.25, *p* = 0.029], while this was not found for the controls [*r*(77) = 0.10, *p* = 0.374; difference: *z* = 2.17, *p* = 0.030].

### External Rating of the *Remember Events*

**Figure 2:**
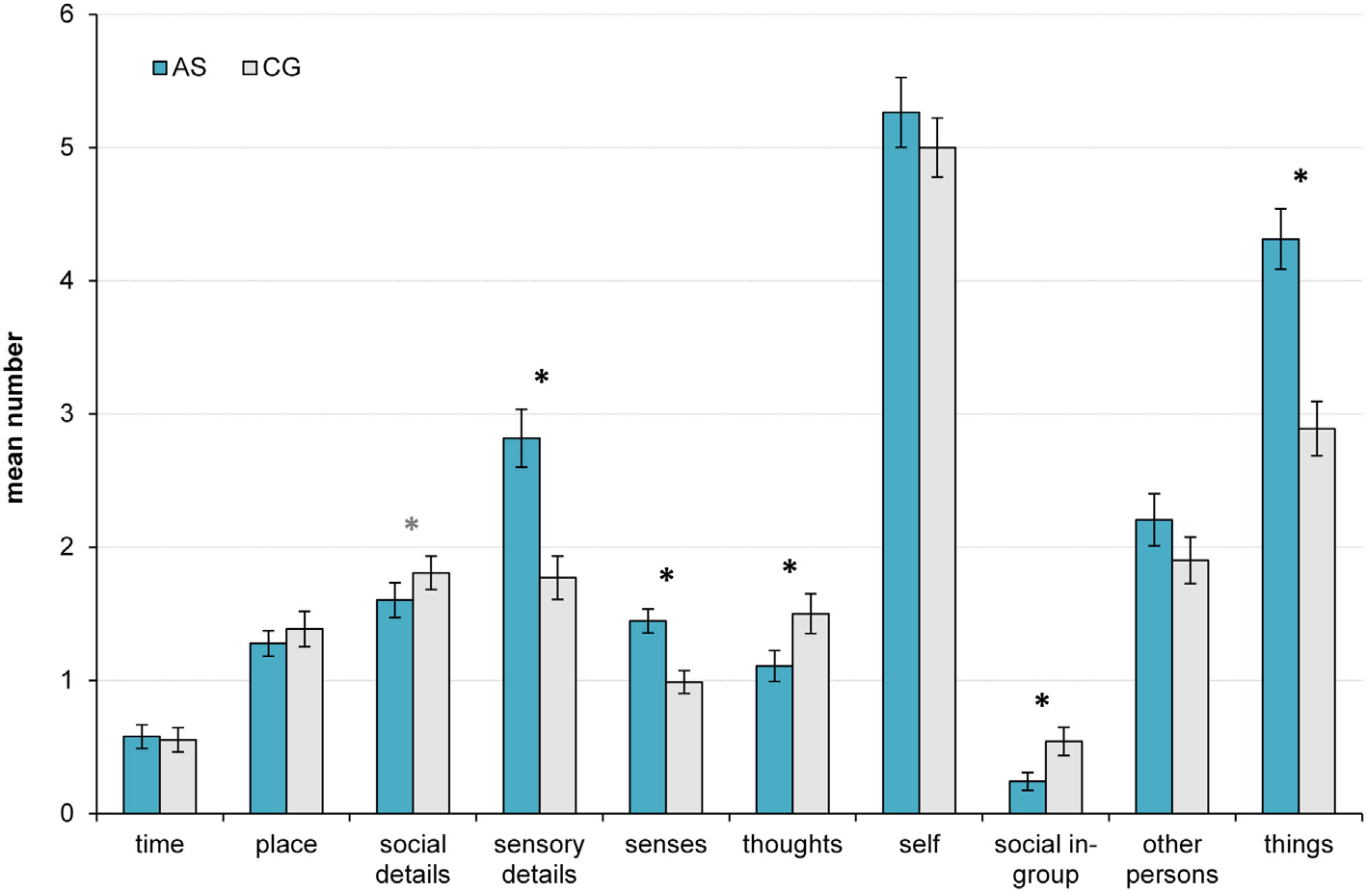
**Mean ratings of different aspects and four word counts (self, social in-group, other persons, things) of *remember events* of participants on the autism spectrum (AS) and matched controls (CG)**. Error bars indicate ± SE; *indicates significant differences between groups (*p* < 0.05), *only significant after exclusion of depression/AD(H)D; for further details, see text.

Overall word count of the descriptions of *remember events* did not differ significantly between groups [AS 40 ± 27, CG 35 ± 14, *F*(3,165) = 1.00, *p* = 0.319, *f* = 0.08; excluding depression/AD(H)D: AS 37 ± 18, CG 34 ± 12, *F*(1,135) = 1.24, *p* = 0.268, *f* = 0.10]. Furthermore, no group reported more fragment memories (AS: 21 fragments, one not clearly relatable, 61 “complete”; CG: 21 fragments, 2 not clearly relatable, 60 “complete”). External ratings by the authors showed a similar pattern compared to the internal self-ratings by the participants. Word count of social in-group words and things differed significantly between groups: in the AS group, less words referring to the social in-group and more words referring to things were used [social in-group: *F*(3,165) = 6.91, *p* = 0.009, *f* = 0.21; things: *F*(3,165) = 24.48, *p* < 0.001, *f* = 0.39]. However, word counts of references to self or to other persons did not differ between groups [self: *F*(3,165) = 0.05, *p* = 0.817, *f* = 0.00; other: *F*(3,165) = 0.92, *p* = 0.340, *f* = 0.08]. More sensory details were reported in the AS group and they described more different senses from which the details derived [details: *F*(3,165) = 17.66, *p* < 0.001, *f* = 0.33, excluding depression/AD(H)D*: F*(1,135) = 13.09, *p* < 0.001, *f* = 0.31; senses: *F*(3,165) = 12.48, *p* = 0.001, *f* = 0.28, excluding depression/AD(H)D: *F*(1,135) = 13.21, *p* < 0.001, *f* = 0.31]. Thoughts and emotions were mentioned less often by autistic participants [*F*(3,165) = 7.78, *p* = 0.006, *f* = 0.22, excluding depression/AD(H)D: *F*(1,135) = 4.70, *p* = 0.032, *f* = 0.19], and also social details were less abundant [*F*(3,165) = 4.37, *p* = 0.074, *f* = 0.14; excluding depression/AD(H)D: *F*(1,135) = 4.52, *p* = 0.035, *f* = 0.18]. Details to time and place, often the measure for specificity, did not differ between groups [time: *F*(3,165) = 0.01, *p* = 0.931, *f* = 0.00; place: *F*(3,165) = 0.19, *p* = 0.668, *f* = 0.03]. Please see also Figure 2.

## Discussion

This study aimed to investigate differences in early childhood memories between people on the AS and controls. We used online questionnaires to better meet the special needs of individuals with autism than it would be possible in a laboratory setting. In addition, in comparison to previous studies on personal autobiographical memory in autism, we asked participants for their earliest *remember* and *know event* without making further restrictions. Based on the intense world theory (21), in line with the expectation of Lyons and Fitzgerald (13), and autobiographical accounts written by people with autism [e.g., Ref. (11, 12)], we hypothesized that people on the AS would have earlier autobiographical memories when not being constrained to a specific predefined category or situation. In addition, we were interested in the influence of sensory details and language on early memory formation in autism.

Our results show that participants on the AS remember earlier events and report more sensory details. In addition, the amount of remembered visual details was related to the age of earliest *remember events* in the AS group and supports our prediction of earlier childhood memories in autism and an association of these to sensory processing. Furthermore, the association between more frequent talking about memories and a younger age of the earliest *remember event* in both groups hints also at an influence of language processing on personal autobiographical memory in autism.

Individuals on the AS remembered events from a significantly earlier age in childhood than the CG. The median age of *remember events* in the CG (3.50 years) is comparable to the one found by Bruce and colleagues [3.52 years (36)] supporting the validity of our measure. Furthermore, groups did not differ in the age of their earliest *know event*. While *remember events* reflect own memories, *know events* reflect memories of episodes other people believe to be important. As *know events* will mainly derive from what seems important to non-autistic people, it is not surprising that the groups did not differ in their age of their earliest *know event*. The age between the first *know* and *remember event* has been proposed to correspond to the end of childhood amnesia (36). According to our data, childhood amnesia seems to end earlier in the AS group than in the CG.

Our results of even earlier remembered childhood memories in autism contradict many previous studies, which found mostly deficits in autobiographical episodic memory. It is possible that people with autism performed worse in earlier studies [e.g., Ref. (2, 3)], because the chosen events did not fit with their interests. The same holds true for the study by Tanweer and colleagues (9) who also differentiated between *know* and *remember events*. The authors asked their participants to recollect memories from predefined categories like an event linked to a person, for example. One might find different results when asking for other activities (e.g., less social and more sensory related) due to a higher involvement of people with autism in those activities. In the present study, participants had free choice which events to report, which avoided a possible bias from using predetermined events. Interestingly, when applying a sentence completion test to participants with autism in a study requiring less social contact (letters), Crane and colleagues (42) found no evidence for deficits in autobiographical memory either. These findings indicate that people on the AS do not have an overall deficit in autobiographical episodic memory, but might differ from non-autistic people in what they encode and/or remember. Interestingly, specificity or episodicity is often said to be impaired in the memories of people with autism (9), whereas in the current sample, the AS group reported as many details related to time and place as the controls did. Maybe episodicity is preserved due to higher involvement in these self-selected events compared to predefined ones.

Participants on the AS reported more sensory characteristics of their *remember events* compared to the CG, and they included a greater number of different senses, e.g., sound in addition to visual details. The authors’ external ratings of the descriptions of the *remember events* revealed the same pattern of results as the self-ratings by the participants. Hence, differences in sensory involvement between people with and without autism could be an explanation for the AS group remembering earlier events. Since people with autism show heightened responses to sensory stimulation [e.g., Ref. (15–17, 19)] memory processing *via* sensory features might be enhanced in adults with autism, which would facilitate retrieval of those memories using sensory pathways. Our results support these assumptions and are also in line with the hyperperception proposed by Markram and Markram (21). Even if memories fade similarly over time in both groups, an initially increased attention to and perception of sensory details in the AS group may explain the increased richness and earlier onset of their memories. In addition, earlier memories may also be related to hypermemory, which may be a starting point for future studies.

This is also in agreement with the increased attention to fine detail (43), leading people with autism to remember details of a situation rather than the global setting. However, we do not know whether children with autism encode more sensory details than non-autistic children or differ only in the ability to retrieve those details later. It would be interesting for future studies to compare the amount of encoding of sensory details between children and adults with and without autism. As evidenced by Hilton and colleagues (19), who found relations between atypical sensory responsiveness and social behavior problems in children with autism, sensory inputs must be considered in the development of coping strategies for social problems. Our results suggest that sensory features are also important in memory processes. For this reason and also as sensory perception is atypical in the majority of people with autism, the assessment of patterns of intensity of sensory perception, and consequently the person-specific modulation of sensory inputs might be a useful tool for learning interventions in autism.

We evaluated the memories regarding several social aspects. Participants on the AS reported fewer social interactions and used words indicating group membership such as “we” or “our” less frequently. These results may reflect less engagement or sense of belonging in social situations and consequently fewer reports. This may also explain why some studies using cued recall find poor autobiographical episodic memory in autism, if the cues largely comprise social events or social details. In contrast, memories of individuals on the AS more often referred to things (e.g., toys, furniture, and animals), and descriptions were rich in sensory detail.

Remarkably, there are no differences in the number of self- or other references between groups, so our results do not support an increased self-focus in people on the AS. Group differences, however, may simply be caused by differences in narrative styles and also be influenced by the experience that non-autistic people are often not able to relate to the different perceptions, emotions, and thoughts experienced by autistic people, which may cause the latter group to keep these mental states to themselves.

Childhood amnesia refers to the phenomenon that early childhood memories are lost, possibly due to fundamental changes in the encoding of events within the first 4 years of life. One theory of childhood amnesia is based on the predication that children are motivated by different goals than adults. Such differences in motive configuration lead to different levels of involvement in a particular situation, which in turn influences which memories are encoded (20). If these processing levels or motivation goals are established very early or are not subject to fundamental changes in autism, this different, not-integrative processing may be the reason for the recall of earlier memories.

Another factor that influences the age of the earliest childhood memory is the use of language. While people with autism with typical language development can use language in addition to sensory cues to encode memories, people with autism with delayed language development are possibly limited to just sensory cues for encoding. As retrieval is easier if more pathways are used at the same time (44), one might speculate that people with autism with typical language acquisition can more easily retrieve early autobiographical memories than people with autism with delayed language acquisition. The age of language acquisition should be interpreted with caution, since it is usually learnt from relatives or caregivers, and may differ in accuracy between individuals; however, often times, these early milestones in the development of children are very important to relatives, and thus better remembered or even written down. Furthermore, inaccuracies should be distributed equally over individuals of both groups, likely not influencing our results. As elaborative talk was found to facilitate the development of autobiographical memory skills (45), it is possible that talking more often about the *remember event* also improves the ability to remember the event later in life. In agreement with the negative correlation between age of language acquisition and the amount of talking about the earliest remembered memory in the AS group, people on the AS remembered even earlier events when they reported talking more about them. In sum, it seems that language also influences autobiographical memories of people on the AS. For this reason, it would be interesting for further studies to include a greater number of people with autism with language delay and to ask for the rating of language features of the *remember events per se* as well as taking into account the ability of several people with autism to comprehend language without using it verbally.

The present study was conducted online. This procedure has several advantages. Laboratory conditions might not be suitable for many individuals with autism as many of them may find social interactions overwhelming and would choose not to participate, resulting in a selection biased sample. In addition, by conducting the survey online, individuals with and without autism may feel less pressure to report socially desirable events. Interestingly, one of the rare studies that found not only deficits but also strengths in the autobiographical memory of people with autism, was a study in which the memory task was mailed to the participants, allowing them to complete it in their preferred environment instead of a laboratory setting, suggesting a beneficial effect for non-laboratory-based assessments of autobiographical memory in autism (42). Nonetheless, it would be important to compare laboratory and online conditions in reporting early childhood events, although it seems likely that the results of age estimation are comparable, as the paper and pencil study of Bruce and colleagues (36) had similar results in the age of the earliest *remember events* for controls (see above).

The drawback of the online approach is that it is not possible to have a diagnostic interview with the participants. So, despite using the AQ as a reliable screening instrument (37, 39) and asking participants to self-report professional diagnoses, it is possible that not all people who are included in the AS group have autism and not all people in the CG were non-autistic. However, any potential misclassification of participants in this study would reduce differences rather than accentuating them. Therefore, we assume that our result holds true especially in a more controlled sample. The impossibility of verifying the memories is a limitation of the study. However, reports of false memories are possible for everyone, including relatives. Additionally, this would raise the problem of different involvement of children and adults as well as non-autistic and autistic people, as discussed above. Since a substantial number of first memories in our study would have required verification from non-family members, such as kindergarten teachers, checking every single event is not possible and we would have had to restrict the memories (e.g., situations also experienced and remembered by another available person), which would have led to a biased sample. A further limitation is that only people on the AS who we see as high-functioning and who were able to report their memories were included in the study. So, it is not clear whether these results would be the same for people with autism who are not able to report their perception in a way for others to understand. Thereby, a new problem emerges, would then the results be due to communication difficulties or altered experience.

In a nutshell, people on the high-functioning AS do not seem to have an overall deficit in personal episodic memory, instead remembering autobiographical memories from an even earlier age in childhood compared to a CG. This could be due to a more sensory-based form of memory processing in the AS group, resulting in an improved retrieval of sensory details in adulthood. The findings from the present study are in agreement with autobiographies from people with autism [e.g., Ref. (11, 12)], the work by Hilton and colleagues (19), Hochhauser and Engel-Yeger (46), and the intense world theory of autism by Markram and Markram (21), who all assume that sensory features play a major role in autism. Furthermore, also language seems to influence autobiographical memory in autism, as in the AS group an earlier use of language was associated with talking more about the *remember events* and remembering earlier ones. Assessing the pattern of intensity of sensory perception is a potentially useful tool for understanding the heterogeneity of symptoms in autism, developing effective interventional methods, and also of course in day-to-day interactions with people with autism.

## Author Contributions

VZ designed and carried out the study, DM and SS participated in the rating of the events, and PK helped with coordination. All authors contributed to the statistical analysis, interpretation of the data, drafting the manuscript, provided critical revisions, and read and approved the final version of the manuscript.

## Conflict of Interest Statement

The authors declare that the research was conducted in the absence of any commercial or financial relationships that could be construed as a potential conflict of interest.

## Footnotes

^1^Translations available here: http://www.autismresearchcentre.com/arc_tests

## Appendix

- Examples of *Remember Events* of Individuals on the Autism Spectrum (Translation by the Authors)
  - My earliest memory is that we went on a ferry. The seats were red, and I was still so small that I could cross lie on the bench. I did not feel well.
    [this is the one used in the abstract]
  - The fresh peach split into two halves which I ate in kindergarten. Velvety skin, a special texture of the pulp. How the core was furrowed. The orange color of the pulp, the reddish skin.
  - Visiting my granny just before she died. At the bedroom door, my mother advised my sister and me to be good and quiet. For the funeral, my mother bought me some dark blue corduroys.
  - My earliest memory is about a vacation in southern France. We had a vacation house which was called “the little house at the ocean.” We went there all the way in the blue Fiat. My brother was still a baby. The sand on the beach was so hot that I burned my feet.
- Examples of *Remember Events* of Controls (Translation by the Authors)
  - On my fourth birthday, I had chickenpox, which is why my children’s party was canceled. Then, my parents drove to a lake with me.
  - In kindergarten, another child received a worksheet and was praised for its handling. I wanted to please the kindergarten teacher and took a sheet from the desk and filled it in. Unfortunately, I was scolded for it.
  - I remember the first holiday with my family. I was 3 years old. We flew to Crete and my brother and I got an inflatable airplane toy on the plane.
  - I sat on the edge of the bed and “read” to my parents from a book, though I could not actually read.
